# Low Molar Mass Dextran: One-Step Synthesis With Dextransucrase by Site-Directed Mutagenesis and its Potential of Iron-Loading

**DOI:** 10.3389/fbioe.2021.747602

**Published:** 2021-09-09

**Authors:** Tingting Wang, Zhiming Jiang, Yiya Wang, Hao Wu, Yan Fang, Weiliang Dong, Bin Wu, Jiangfeng Ma, Min Jiang

**Affiliations:** State Key Laboratory of Materials-Oriented Chemical Engineering, College of Biotechnology and Pharmaceutical Engineering, Nanjing Tech University, Nanjing, China

**Keywords:** GH70 family, dextransucrase, iron dextran, low molar mass dextran, site-directed mutagenesis

## Abstract

Iron dextran is a common anti-anemia drug, and it requires low molar mass dextran as substrate. In this work, we selected 11 amino acid residues in domain A/B of DSR-MΔ2 within a 5-angstrom distance from sucrose for site-directed mutagenesis by molecular docking. Mutation of Q634 did not affect the enzyme catalytic activity, but showed an obvious impact on the ratio of low molecular weight dextran (L-dextran, 3,000–5,000 Da) and relatively higher molecular weight dextran (H-dextran, around 10,000 Da). L-dextran was the main product synthesized by DSR-MΔ2 Q634A, and its average molecular weight was 3,951 Da with a polydispersity index <1.3. The structural characterization of this homopolysaccharide revealed that it was a dextran, with 86.0% α(1→6) and 14.0% α(1→4) glycosidic linkages. Moreover, L-dextran was oxidized with NaOH and chelated with ferric trichloride, and an OL-dextran-iron complex was synthesized with a high iron-loading potential of 33.5% (w/w). Altogether, mutation of amino acids near the sucrose binding site of dextransucrase can affect the chain elongation process, making it possible to modulate dextran size.

## Introduction

Dextran is a homopolysaccharide generally composed of α-1,6-glycosidic linkage and occasionally branched with α-1,2, α-1,3, and α-1,4-glycosidic linkages, and it is mainly synthesized with sucrose as substrate by dextransucrase from *Streptococcus, Lactobacillus,* or *Leuconost*oc (Leemhuis, et al., 2013). Due to its favorable biocompatibility, low toxicity, and the existence of a large number of hydroxyl groups in their structure, dextran and its derivatives have been widely studied in food, medicine, cosmetics, and other areas (Ates, 2015; Ghimici and Nichifor, 2018; Chen et al., 2020). Among them, low molar mass dextran has received wide attention due to its biological activities including its antioxidant, immunomodulatory, and antimicrobial properties (Moreno-Mendieta et al., 2017; Xia et al., 2021).

Iron dextran is a complex synthesized with low molar mass dextran and ferric oxyhydroxide, and it is a common anti-anemia drug that is mainly used to treat iron-deficiency anemia (Liu et al., 2019). However, most of the current commercial production of iron dextran utilizes dextran with an average molecular weight between 7,000 and 24,000 Da as substrate, and high molecular weight dextran may induce allergic reactions (Jahn et al., 2011; Kalra and Bhandari, 2016). Although dextran with an average molecular weight between 3,000 and 6,000 Da has recently been recognized to be the best substrate for producing high-quality iron dextran, no enzymatic process has yet been established for the conversion of substrate sucrose directly to this low molar mass dextran. To date, enzymatic conversion generally synthesizes high molar mass dextran, and further degradation is required to obtain different molar mass dextrans by enzymolysis or acidolysis. During the degradation process, impurities were introduced and the yield of target dextran product based on substrate sucrose was decreased (Gan et al., 2014). Thus, exploring a one-step enzymatic method to produce dextran of 3,000–6,000 Da has practical significance in the commercial production of iron dextran.

Dextran is at present industrially synthesized mainly by a reaction catalyzed by dextransucrase, which synthesize dextran by hydrolysis of the glycosidic bond of sucrose and uses the released energy to transfer d-glucopyranosyl residues to the growing polymer, concomitantly releasing fructose. These enzymes belong to the Gycosyde hydrolase 70 (GH70) family, and dextransucrase adopts a U-shaped fold that results in an organization of 5 domains with domain A as the catalytic domain containing a (β/α)_8_-barrel (Vujičić-Žagar et al., 2010). The amino acid residues of the catalytic triad (aspartic acid nucleophile, glutamate acid-base catalyst involved in the formation of β-D-glucosylate, and the third aspartic acid acting as a transition state stabilizer) are also located in the deep pocket of domain A (Vujicić-Zagar and Dijkstra, 2006; Ito et al., 2011). Thus, mutation of these amino acid residues will inactivate the enzymatic activity (Vujičić-Žagar et al., 2010). It has been previously reported that site-directed mutagenesis of glucosylhydrolases alters the relative balance of the three reactions, including synthesis of dextran, hydrolysis, and the synthesis of oligosaccharides (Leemhuis et al., 2012).

We recently expressed a dextransucrase DSR-MΔ2 in *Escherichia coli*, the sole enzyme that naturally produces only low molar mass dextrans from sucrose, which has been reported by Claverie et al. (2017). However, the products synthesized by DSR-MΔ2 were not uniform, producing low molecular weight dextran (L-dextran, 3,000–5,000 Da) and relatively higher molecular weight dextran (H-dextran, around 10,000 Da) simultaneously. Thus, we performed molecular docking with substrate sucrose to DSR-MΔ2, and constructed 11 point mutations to determine whether the size or structure of dextran could be modified. Of these, Q634A displayed significant effects on dextran synthesis and produced a high proportion of L-dextran. Furthermore, NaOH-oxidizing modification and iron chelation were carried out with this L-dextran to analyze its iron-loading potential.

## Materials and Methods

### Strains and Plasmids, Media, and Conditions

The DSR-MΔ2 gene was inserted into plasmid pET-28a, and the mutation of plasmid pET28a-*dsrMΔ2* was constructed by using the primers in Supplementary Table S1 in the Supporting Information. *E. coli* BL21 (DE3) was used as the host for the expression of enzyme DSR-MΔ2 and its variants.

*E. coli* strains were cultured in Luria-Bertani (LB) medium (10 g/L tryptone, 5 g/L yeast extract, and 10 g/L NaCl) with 100 μg/ml of kanamycin at 37°C for growth and at 25°C for enzyme expression with shaking at 200 r/min. IPTG (0.05 mM) was added to the fermentation broth when optical density at 600 nm (OD_600_) reached 0.6–0.8 for a 24-h induction, and then cells were harvested by centrifugation, resuspended in lysis buffer (20 mM phosphate sodium buffer, pH 7.0), and disrupted by sonication. After centrifugation, recombinant enzymes were recovered in the soluble fraction of the crude cell extract, and it was then purified by ammonium sulfate precipitation and Sepharose 6B gel filtration chromatography (Gan et al., 2014).

### Molecular Modeling and Docking

The structure of dextransucrase DSR-MΔ2 was downloaded from the PDB database (PDB entry: 5LFC), and the substrate sucrose structure was extracted from the crystal structure of DSR-MΔ2 E715Q (PDB entry: 5O8L) and docked into the active site of dextransucrase DSR-MΔ2. The docking region was defined as encompassing the amino acid residues of the catalytic triad in the pocket of domain A/B. All images were visualized through the software PyMol™ 2.2.0.

### Enzymatic Reaction and Product Preparation

The enzymatic activity of the mutant dextransucrases was determined by the dinitrosalicylic acid method (Bejar et al., 2013), and 1 unit was defined as the amount of enzyme that catalyzed the formation of 1 mmol of fructose and/or glucose per minute from 584 mM sucrose in 50 mM sodium acetate buffer at pH 5.8.

Enzymatic reactions were performed at 30°C on 200 g/L sucrose in 50 mM sodium acetate buffer at pH 5.8 for 5 h, using 2 units of enzyme per liter of reaction mixture, and halted by 30 min of incubation at 100°C. Samples were freeze-dried and stored at −20°C until further analysis.

### Separation and Purification of Dextran

Purification of dextran was performed by adopting membrane-separation technology. The raw dextran solution was passed sequentially through a microfiltration membrane (MOF 205) and two ultrafiltration membranes (S-UF 5.0 K, S-UF 1.0K) with molecular weight cut-offs of 5,000 Da and 1,000 Da, respectively. The operative conditions were maintained at a temperature <40°C and an operating pressure <0.3 MPa. The target product was present in the trapped fluid of the S-UF 1.0 K ultrafiltration membrane.

### Concentration Calculation of L-Dextran

Peak area of gel permeation chromatography (GPC) can indicate the concentration of polymer (Wang et al., 2020). In this study, to analyze the concentration of L-dextran, the purified L-dextran from enzymatic reaction was firstly dried to constant weight as the L-dextran standard. GPC was used to analyze the L-dextran samples, and we have constructed the curve (Supplementary Figure S1). The concentration *Y* (g/L) of L-dextran was calculated by the peak area (*X*) with the formula: *Y* = 0.17*X* + 0.0029.

### Molecular-Size Analysis of Dextran

The molecular size of the dextran was determined using an Ultrahydrogel 250 column (7.8 × 300 mm) and Ultimate 3000 HPLC system using a refractive index detector. The temperature was maintained at 40°C, and high-purity water with a flow rate of 0.5 ml/min was used as an eluent. Dextran standards of different molecular weights (Shanghai yuanye Bio-Technology Co., Ltd, T3 (3,000 Da), T5 (5,000 Da), T7 (7,000 Da), T9 (9,000 Da), T10 (10,000 Da)) were used as standards to calibrate dextran size. The standards and freeze-dried dextran samples were dissolved to 1 g/L, and 20 μL was applied for molecular-size analysis. The molecular size *M* was calculated by the retention time *tR* with the formula: *M* = −2069.2 *tR* + 37,985.

### Structural Analysis of Dextran

The FT-IR spectra were measured using a Thermo Fisher IS5 in the scanning range of 400–4,000 cm^−1^. The dextran film was prepared with dried potassium bromide (KBr) powder in a ratio of 1:100 and pressed into a 1-mm pellet prior to scanning. For NMR spectroscopy, a sample of approximately 20 mg of purified dextran was dissolved in 99.9% D_2_O. ^1^H chemical shifts were referenced to internal D_2_O (4.8 ppm at 30°C). The type and ratio of the glycosidic bonds were analyzed according to the ^1^H and ^13^C NMR spectra (Miao et al., 2014).

### Preparation of Carboxyl-Modified Dextran and Iron Dextran

A dextran solution containing 20% (w/w) dry solid was prepared, and the pH was adjusted to 12 with a 20% (w/w) NaOH solution. The reaction mixture was maintained at 65°C for 4 h, and after NaOH oxidation, a 40% (w/w) FeCl_3_ solution was added to the solution in a slow drip with stirring, and the pH of the solution was adjusted to 6.5–7.0. Heating and stirring were maintained for another 6 h. Finally, an iron dextran complex was obtained by dialysis treatment and freeze drying.

To determine the iron content in OL-dextran–iron complex, phenanthroline method was performed (Fang et al., 2021). Two samples were prepared, that is, one sample was dissolved with deionized water to analyze the free iron (C1) and another sample was dissolved with 20% hydrochloric acid to disperse the iron in the complex so as to analyze the total iron (C2). Then, the iron content in the OL-dextran–iron complex equals C2 minus C1.

## Results and Discussion

### Selection of Mutation Sites

**FIGURE 1:**
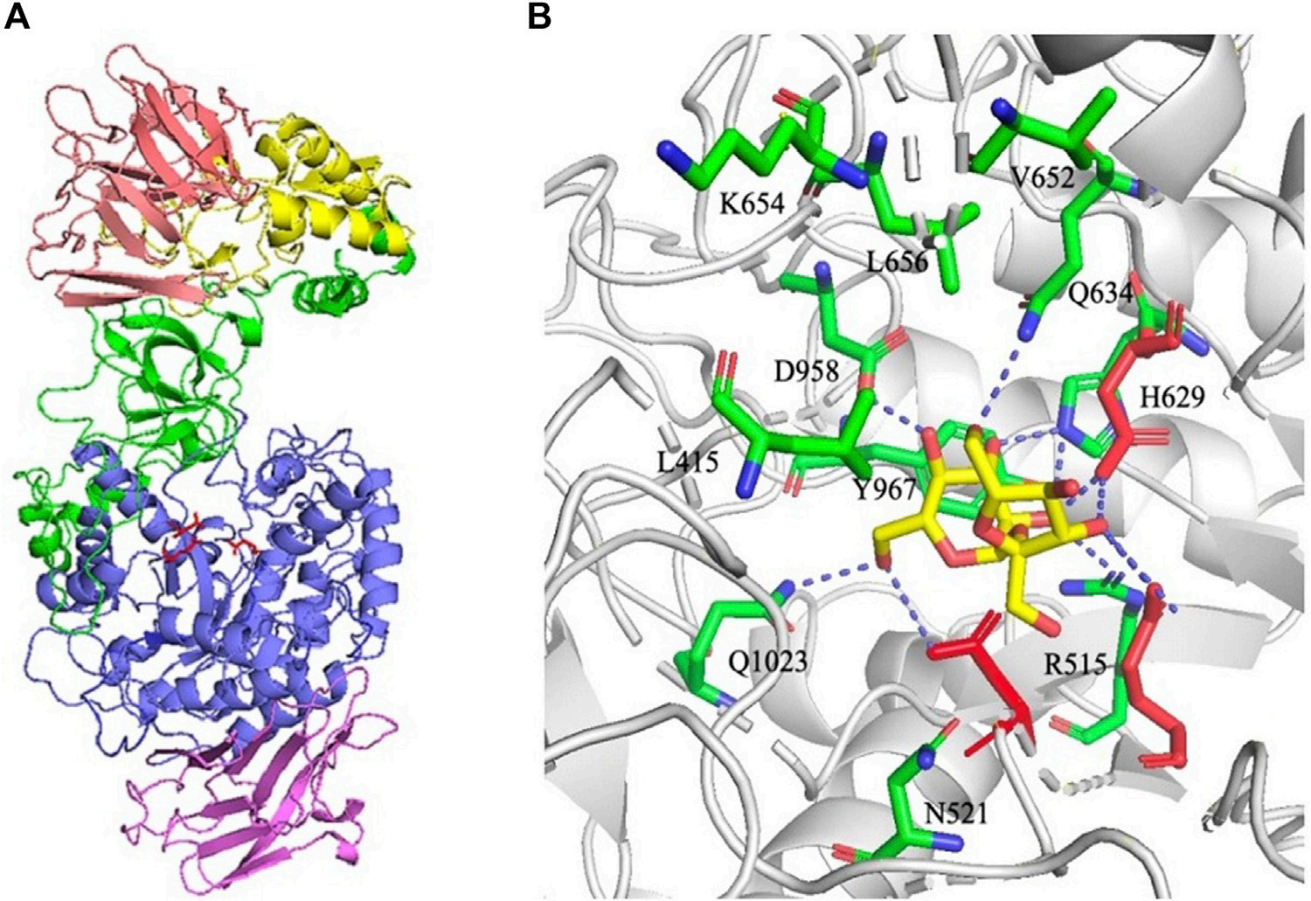
**(A)** Structural organization of DSR-MΔ2 (PDB entry: 5LFC). Domains A, B, C, 4, and 5 are colored in blue, green, purple, yellow, and pink, respectively. Catalytic residues Asp517 (nucleophile), Glu555 (acid/base), and Asp630 (transition state stabilizer) are shown with red sticks. **(B)** Structure of DSR-MΔ2 in complex with sucrose visualized through the software PyMol™ 2.2.0. Eleven amino acids within a 5-angstroms distance from sucrose are marked in green sticks. Catalytic residues Asp517, Glu555 and Asp630 are shown with red sticks, and substrate sucrose was shown with yellow sticks. Hydrogen bonding interactions are shown as blue dashed line.

Previously, dextransucrase DSR-MΔ2 was constructed by truncation of the glucan binding repeats in DSR-M, and the molecular weight of the product was significantly decreased. In addition, individual amino acids including Tyr180 and Tyr264 in domain V implicated directly in the glucan binding pockets and Trp624 in domain B near substrate binding site were selected to construct mutants, and the final size of dextran catalyzed by these mutants were further decreased (Claverie et al., 2017; Claverie et al., 2019). Thus, modification of DSR-MΔ2 by mutation of amino acids in the glucan binding pockets or near sucrose might regulate the chain-length of the product. Here, we firstly performed molecular docking with substrate sucrose docked into the active site of DSR-MΔ2 using the software package AutoDock 4.2. As shown in Figure 1B, substrate sucrose is marked by magenta sticks, and 11 amino acid residues (L415 and Q1023 in domain B, and R515, N521, H629, Q634, V652, K654, L656, D958 and Y967 in domain A) within a 5-angstrom distance from sucrose are marked by green sticks. In order to study how these amino residues regulated the synthesis of dextran, site-directed mutagenesis was carried out with the aim of obtaining a series of mutants.

R515 was substituted for aspartic acid to determine the effects of acidic and basic residue. N521 was substituted with tyrosine to detect function when increasing the side chain and introducing an aromatic ring. H629 was substituted with aspartic acid to investigate results after reducing the side chain and changing the basic residue to an acidic one. Q1023 was substituted with lysine to determine the effects of the removal of the amide group on the side chain. The remaining 7 amino residues were substituted for alanine to determine the effects of different groups on their side chains.

### Activity Analysis of Mutant Enzymes

**FIGURE 2:**
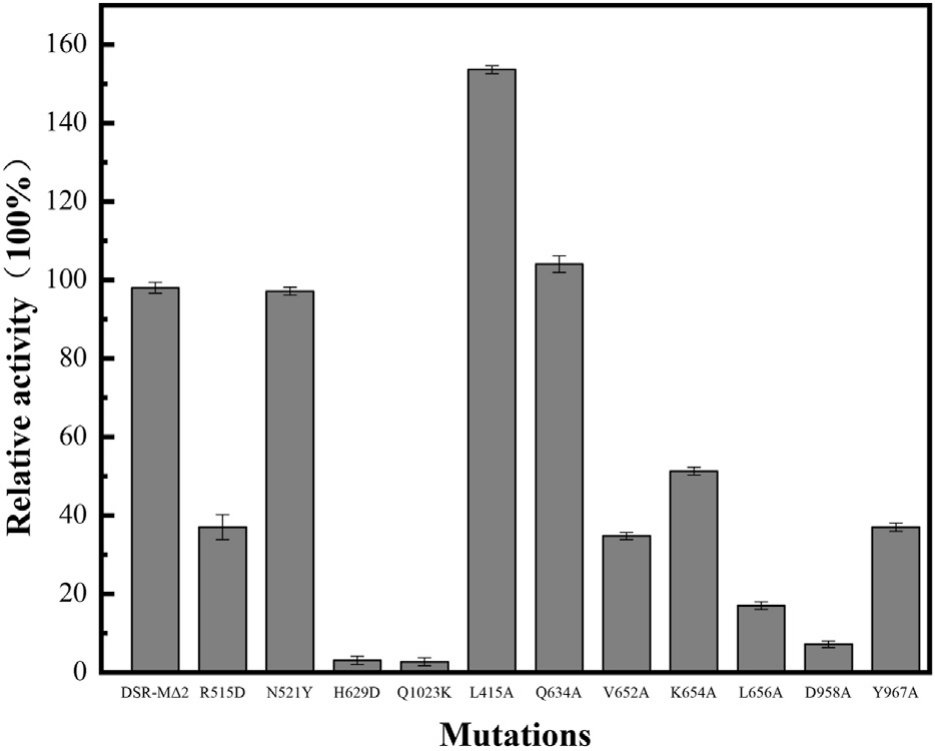
Enzyme activity of DSR-MΔ2 and its eleven mutants.

We performed activity assays with enzyme DSR-MΔ2 and its variants as shown in Figure 2, and observed that mutants L415A possessed higher enzyme activity than DSR-MΔ2, and mutation of Q634 had the similar activity. In the case of N521Y, there was only a slight loss of activity, and mutants R515D, V652A, K654A, L656A, and Y967A exhibited enzyme activities of approximately 10–50% with respect to the original enzyme. The other mutants (H629A, D958A, and Q1023K) manifested activities lower than 5% that of DSR-MΔ2, indicating that these three amino acid residues were important residues for dextran synthesis. This is in agreement with the severe loss of activity with mutation of W717 in domain A of DSR-MΔ2, which reduced >99% activity (Claverie et al., 2019). Also, drastic loss of activity with only 4, 3, 2 and 0.5% residual activity were found in the mutants of *Lactobacillus reuteri* 121 reuteransucrase GTF-A by the mutation of S663Y, SEV663YDA, SEV663NNS and SEVQTVI663KGVQEKV, respectively (Moulis et al., 2006).

### Distribution of Dextran Produced by the Mutant Enzymes

**FIGURE 3:**
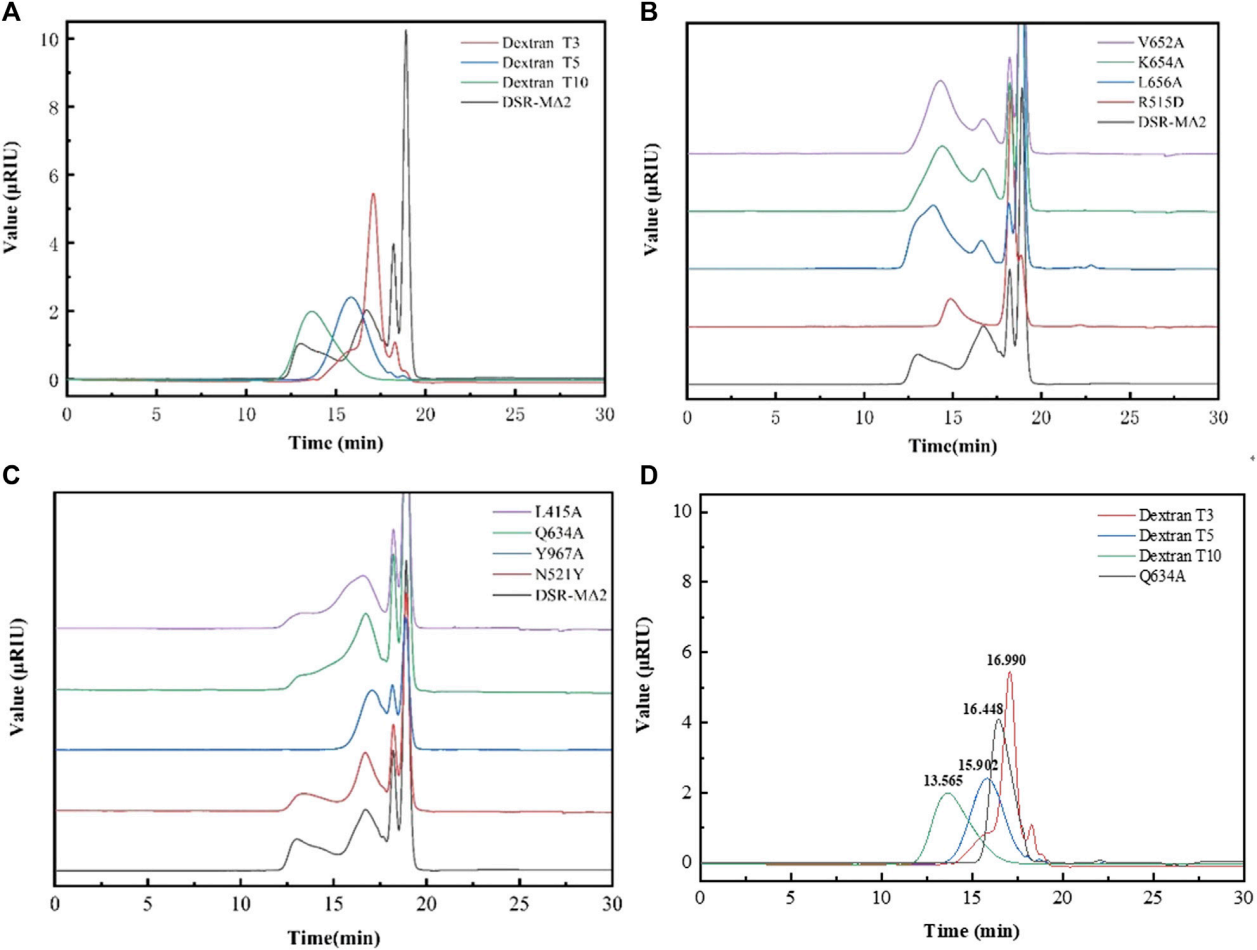
Analysis of the molecular weight of dextrans synthesized by DSR-MΔ2 and its variants from sucrose. **(A)** Dextran synthesized by DSR-MΔ2. T3, T5 and T10 are the standards with 3,000 Da, 5,000 Da and 10,000 Da; **(B)** Dextran synthesized by DSR-MΔ2 mutants with higher ratio of H-dextran; **(C)** Dextran synthesized by DSR-MΔ2 mutants with higher ratio of L-dextran; **(D)** Purified dextran synthesized by Q634A. Also, T3, T5 and T10 are the standards with 3,000 Da, 5,000 Da and 10,000 Da.

As shown in Figure 3A, dextran showing a bimodal distribution was synthesized with DSR-MΔ2 from sucrose. The low molecular weight of dextran (L-dextran) was 3,000–5,000 Da, and that of the higher one (H-dextran) was approximately 10,000 Da. As 3,000–6,000 Da dextran was the best substrate to synthesize iron dextran, we wanted to enhance the proportion of L-dextran. Under the experimental conditions, the molecular weights and distribution of dextran changed markedly with different mutants. Mutants L656A, K654A, and V652A produced a relatively higher proportion of H-dextran compared with DSR-MΔ2 (Figure 3B). R515D synthesized dextran with only one peak, but the average molecular weight was higher than L-dextran, at ∼5,000–10,000 Da. Mutants Y967A, Q634A, N521Y, and L415A produced relatively more L-dextran than DSR-MΔ2, and mutant Q634A expressed the highest synthesis ratio of L-dextran to H-dextran, with only a small amount of the latter (Figure 3C). These results were hence in agreement with that the interaction of amino acids in domain A/B is critical for not only the enzymatic rate constant but also the final product distribution (Moulis et al., 2006; Claverie et al., 2019).

In addition, Q634A produced 84.5 g/L L-dextran, achieving 84.5% of the theoretical maximum yield. Then, we purified the raw dextran solution catalyzed with Q634A to obtain L-dextran by a microfiltration membrane and two kinds of ultrafiltration membranes. As a result, the target L-dextran exhibited a single peak with a recovery yield >80% (data shown in Supplementary Table S2), indicating that this separation method was suitable for purifying L-dextran (Figure 3D). By calculation, its average molecular weight was 3,951 Da, with a polymer dispersity index (PDI) < 1.3, which is rather lower relative to the Chinese Standard (PDI <2.0).

### Linkage Composition Analysis of L-Dextran Synthesized by Mutant Q634A

**FIGURE 4:**
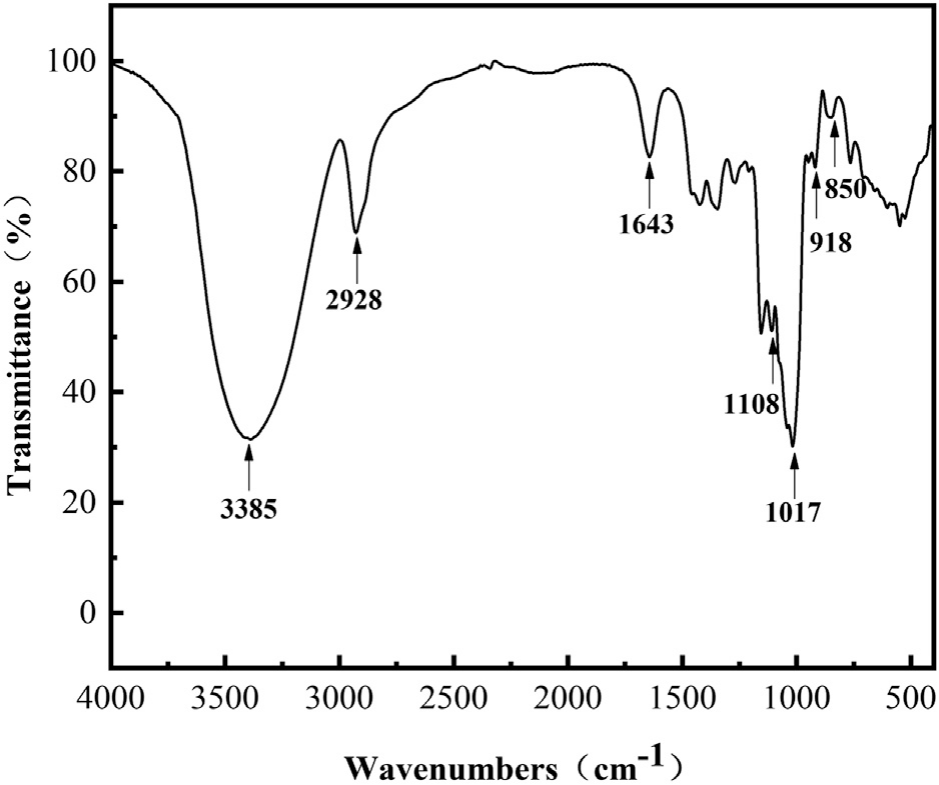
FT-IR spectra of purified L-dextran synthesized by mutant Q634A.

The FT-IR spectra (Figure 4) in the range of 4,000–500 cm^−1^ provides information on the functional groups, monomeric units, and linkages. Thereinto, the absorption peaks at 3,385 cm^−1^ and 2,928 cm^−1^ were assigned as the stretching vibrations of the–OH and–CH/CH_2_ groups of the sugar ring, respectively (Yan et al., 2018). The peak at 1,643 cm^−1^ was due to the C=O group of the sugar hydrate (Li et al., 2016). The absorption peaks at 850 and 918 cm^−1^ indicated the existence of α-glycosidic bonds (Capek et al., 2011; Rao and Goyal, 2013). The bands at 1,017 cm^−1^ and 1,108 cm^−1^ are present in polysaccharide with α-(1, 6) and α-(1, 4) linkages and can be considered as a characteristic for the type of interunit link (Majumder et al., 2009). The spectrum indicated that L-dextran was similar to the fingerprint bands of dextran, and its structure was further confirmed by ^1^H and^13^C NMR analysis.

**FIGURE 5:**
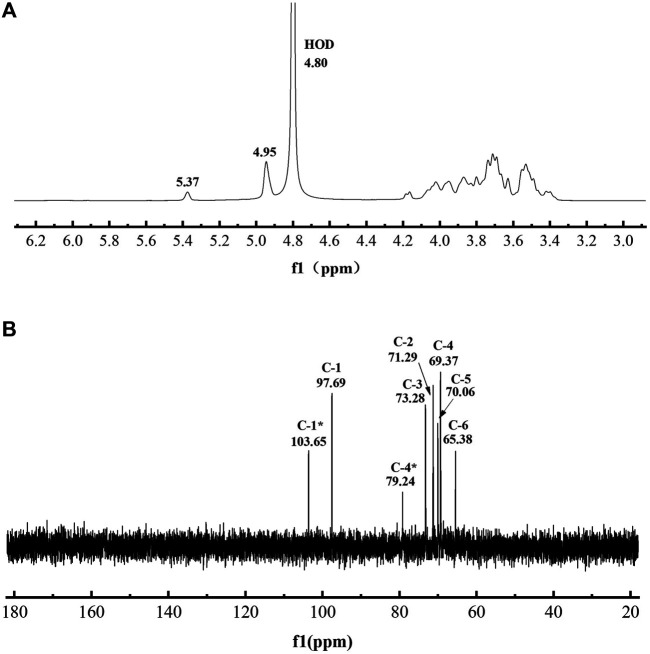
The spectra of purified L-dextran synthesized by mutant Q634A. **(A)** 1H NMR spectra **(B)** 13C NMR spectra.

The chemical structure of L-dextran analyzed by the ^1^H and ^13^C NMR are shown in Figure 5. The positions of anomeric signals at 4.95 and 97.69 ppm in ^1^H NMR and ^13^C NMR spectra are characteristic of α-1,6-glucosidic linkages whereas signals at 65.38 in ^13^C NMR spectra are characteristic of 6-substituted glucopyranose residues (Bashari et al., 2013). The positions of anomeric signals at 5.37 and 103.65 ppm in ^1^H NMR and ^13^C NMR spectra are characteristic of α(1→4) branching whereas signals at 79.24 in ^13^C NMR spectra are characteristic of 4-substituted glucopyranose residues (Majumder et al., 2009; Miao et al., 2017; Rosca et al., 2018). Based on the integration analysis of ^1^H NMR, it was shown that this L-dextran had 86.0% α(1→6) main chain and 14.0% α(1→4) branching. The ratio of the α(1→6) glycosidic bond was lower than that of dextran synthesized by DSR-MΔ2. This indicates that part of the glucosyl residues from sucrose was incorporated into L-dextran as α(1→4) linkage.

### Iron-Chelating Ability Analysis of L-Dextran Produced by Mutant Q634A

**FIGURE 6:**
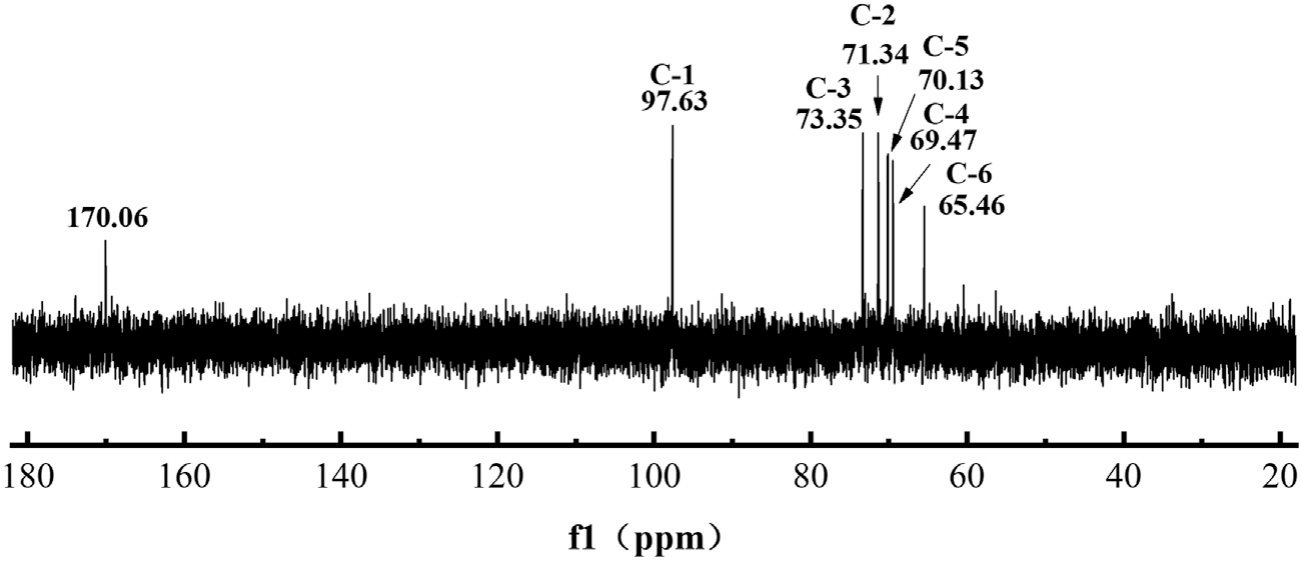
13C NMR spectra of NaOH-modified L-dextran synthesized by mutant Q634A

To analyze the iron-loading potential of L-dextran, we performed NaOH-oxidizing modification and iron chelating. As shown in Figure 6, the oxidized L-dextran (OL-dextran) had a new peak of gluconic acid at ∼170 ppm, along with a diminished peak intensity at 97.63 ppm. These results showed that NaOH not only modified the hydroxyl group at C1 of anhydroglucose to the carboxyl group but also modified other positions of the sugar ring (Shi et al., 2013). In addition, the α(1→4) branching anomeric signals at 103.65 and 79.24 ppm in ^13^C NMR spectra disappeared, which indicated that the α(1→4) linkages were hydrolyzed.

**FIGURE 7:**
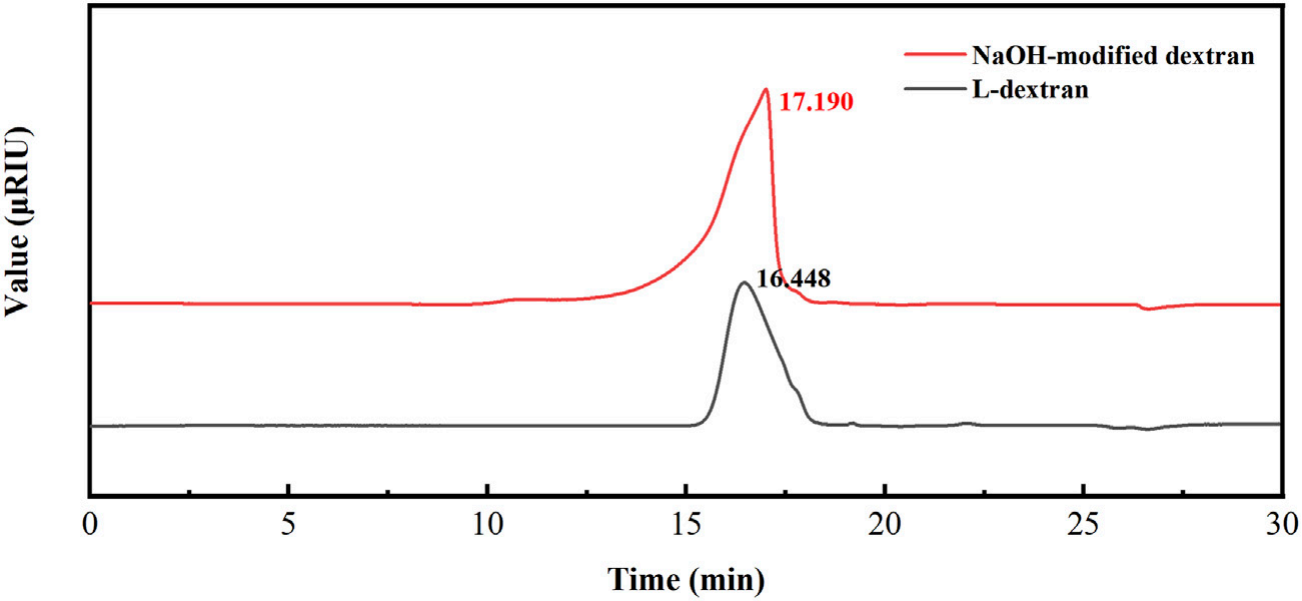
Molecular size distribution of L-dextran and NaOH-modified L-dextran.

From the GPC results in Figure 7, we found the retention time of OL-dextran was prolonged, certifying that the NaOH treatment caused depolymerization. The molecular weight of L-dextran decreased from 3,951 to 2,415 Da. Calculating with the 14% α(1→4) and 86.0% α(1→6) glycosidic bonds of L-dextran, the decreased molecular weight of 1,536 Da should be attributed to entire depolymerization of α(1→4) with partial depolymerization of α(1→6) glycosidic bonds. It has been reported that polysaccharides, specifically those with 1,3-, 1,4-, or 1,6-glycosidic bonds, can be degraded by alkalis (Chen et al., 2018).

**FIGURE 8:**
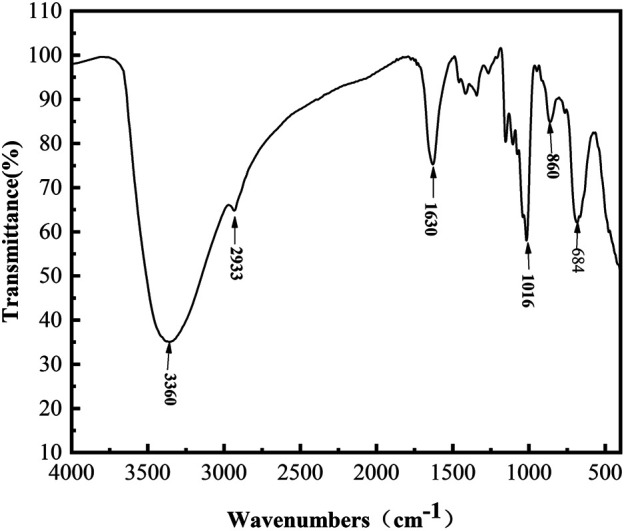
FT-IR spectra of OL-dextran–iron complex.

After iron chelation, an OL-dextran–iron complex was synthesized, and its FT-IR spectrum produced signals at 860 cm^−1^ and 684 cm^−1^ (Figure 8), which indicated that the iron core in this OL-dextran–iron complex was a polymerized β-FeOOH structure (Shi et al., 2013). In fact, the iron core of most iron-dextran complexes and the iron pyroxene (a naturally occurring mineral, β-FeOOH) exhibit very similar or even identical structures (Chen and Huang, 2020). Moreover, the results of displacement iodometry showed that the iron content of OL-dextran–iron was 33.51% (w/w), higher than the 25% (w/w) requirement in Chinese Standards and European Standards, supporting that less substrate dextran will be needed to produce the same mass of iron product.

## Conclusion

Low molar mass dextran was preferred candidate for the production of iron dextran, which is a common anti-anemia drug. Dextransucrase catalyzes the production of dextran using sucrose as sole substrate, and DSR-MΔ2 is one of the dextransucrases that can directly catalyze the synthesis of low molecular dextran. However, the products synthesized are not uniform, synthesis of L-dextran and H-dextran simultaneously. Herein, we constructed and tested 11 point mutations within a 5-angstrom distance from sucrose, and of these, we found that the substitution of Q634 by alanine exerted an obvious impact on the ratio of L-dextran to H-dextran, mainly with L-dextran of 3,951 Da with PDI <1.3. Structural identification revealed that the product was a type of dextran, with 86.0% of α(1→6) and 14.0% of α(1→4) glycosidic linkages. The ratio of α(1→6) glycosidic bond was lower than that for dextran synthesized by the parental DSR-MΔ2, and some of the glucosyl residues from sucrose were incorporated into dextran in an α(1→4) linkage. L-dextran was further oxidized with NaOH and chelated with iron, and the OL-dextran–iron complex was synthesized with a structure of polymerized β-FeOOH, which has a higher iron-loading potential of approximately 33.5% (w/w). In conclusion, we provided a one-step enzymatic method to produce dextran of 3,000–6,000 Da, and it has practical significance in the commercial production of iron dextran.

## Data Availability

The raw data supporting the conclusion of this article will be made available by the authors, without undue reservation.
