# Supplementary material for: Low Molar Mass Dextran: One-Step Synthesis With Dextransucrase by Site-Directed Mutagenesis and its Potential of Iron-Loading
Source: Front Bioeng Biotechnol. 2021 Sep 9;9:747602. doi: 10.3389/fbioe.2021.747602 (PMC8458854; doi:10.3389/fbioe.2021.747602)
Supplement: Supplementary file 1 [file DataSheet1.docx]

TABLE S1: Primers used to generate DSR-MΔ2 variants

| Primer Name | Nucleotide Sequence | Mutation position |
| --- | --- | --- |
| L415A for | 5'- ctctactaaccatgtgcaaaatgg -3' | Mutation at position 415 |
| L415A rev | 5'- ccattttgcacatggttagtagag -3' |  |
| R515D for | 5'- gatagcattcgaattgacgc-3' | Mutation at position 515 |
| R515D rev | 5'- gcgtcaattcgaatgctatc -3' |  |
| N521Y for | 5'- cgcagtagataatgttgatgc -3' | Mutation at position 521 |
| N521Y rev | 5'- gcatcaacattatctactgcg-3' |  |
| H629D for | 5'- tcgagctcatgatagtgagg-3' | Mutation at position 629 |
| H629D rev | 5'- cctcactatcatgagctcga-3' |  |
| Q634A for | 5'- atagtgaggttgcgacaataattgc-3' | Mutation at position 634 |
| Q634A rev | 5'- gcaattattgtcgcaacctcactat-3' |  |
| V652A for | 5'- cccactgcagataaggctttactgg-3' | Mutation at position 652 |
| V652A rev | 5'- agtaaagccttatcagcagtgg-3' |  |
| K654A for | 5'- ccactgttgatgcggctttactgg-3' | Mutation at position 654 |
| K654A rev | 5'- ccagtaaagccgcatcaacagtgg-3' |  |
| L656A for | 5'- aaggctttagcggctaaggatagtgc-3' | Mutation at position 656 |
| L656A rev | 5'- ctatccttagccggtaaagcctta-3' |  |
| D958A for | 5'- catttttggacgcagtagatac-3' | Mutation at position 958 |
| D958A rev | 5'- gtatctactgcgtccaaaaatg-3' |  |
| Y967A for | 5'- caaaatggttatgcgtttactg-3' | Mutation at position 967 |
| Y967A rev | 5'- cagtaaacgcataaccattttg-3' |  |
| Q1023K for | 5'- cctgatcaactttataatatgcc-3' | Mutation at position 1023 |
| Q1023K rev | 5'- ggcatattataaagttgatcagg-3' |  |

TABLE S2: Dextran yield and flux of each separation process

|  | Yield of dextran (%) | Flux of separation（L/m^2^∙h） |
| --- | --- | --- |
| Microfiltration (MOF 205) | 99.6 | 20.82 |
| Ultrafiltration (S-UF 5.0 K) | 94.3 | 8.80 |
| Ultrafiltration (S-UF 1.0K) | 85.5 | 6.48 |
| Total yield of separation | 80.3 |  |


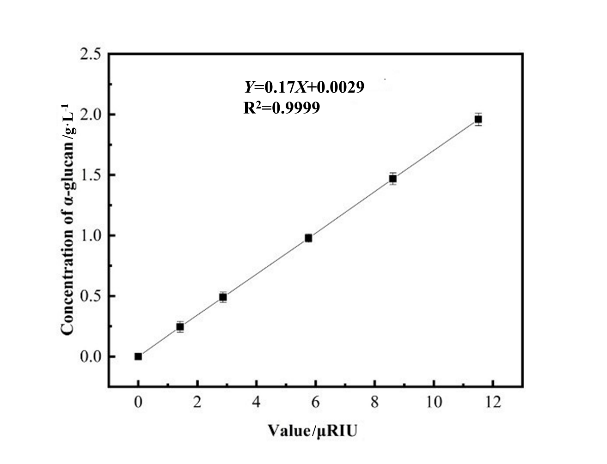


FIGURE S1 Standard curve of L-dextran concentration and peak area. The concentration (*Y*) of L-dextran can be calculated by the peak area (*X*) with the formula: *Y*= 0.17*X*+ 0.0029
